# A practical approach to screening for carbapenemase-producing Enterobacterales– views of a group of multidisciplinary experts from English hospitals

**DOI:** 10.1186/s12879-024-09307-y

**Published:** 2024-04-26

**Authors:** DR. Jenkins, C. Auckland, C. Chadwick, AR. Dodgson, DA. Enoch, SD. Goldenberg, A. Hussain, J. Martin, E. Spooner, T. Whalley

**Affiliations:** 1 University Hospitals of Leicester NHS Trust, Leicester, UK; 2 Royal Devon & Exeter NHS Foundation Trust, Exeter, UK; 3 Bradford Teaching Hospitals NHS Foundation Trust, Bradford, UK; 4 Manchester University NHS FT, Manchester, UK; 5 Cambridge University NHS Foundation Trust, Cambridge, UK; 6 Centre for Clinical Infection and Diagnostics Research, Guy’s and Saint Thomas’ Hospitals NHS Trust, London, UK; 7 University Hospitals Birmingham NHS Foundation Trust, West Midlands, UK; 8 Leeds Teaching Hospitals NHS Trust, Leeds, UK; 9 Royal Wolverhampton NHS Trust, Wolverhampton, UK; 10 Lancashire & South Cumbria ICB, Preston, UK

**Keywords:** Carbapenemase-producing enterobacterales, Antimicrobial resistance, Public health, Healthcare legislation, Guidance, Roundtable meeting

## Abstract

**Introduction:**

Carbapenemase-producing Enterobacterales (CPE) are an important public health threat, with costly operational and economic consequences for NHS Integrated Care Systems and NHS Trusts. UK Health Security Agency guidelines recommend that Trusts use locally developed risk assessments to accurately identify high-risk individuals for screening, and implement the most appropriate method of testing, but this presents many challenges.

**Methods:**

A convenience sample of cross-specialty experts from across England met to discuss the barriers and practical solutions to implementing UK Health Security Agency framework into operational and clinical workflows. The group derived responses to six key questions that are frequently asked about screening for CPE.

**Key findings:**

Four patient groups were identified for CPE screening: high-risk unplanned admissions, high-risk elective admissions, patients in high-risk units, and known positive contacts. Rapid molecular testing is a preferred screening method for some of these settings, offering faster turnaround times and more accurate results than culture-based testing. It is important to stimulate action now, as several lessons can be learnt from screening during the COVID-19 pandemic, as well as from CPE outbreaks.

**Conclusion:**

Further decisive and instructive information is needed to establish CPE screening protocols based on local epidemiology and risk factors. Local management should continually evaluate local epidemiology, analysing data and undertaking frequent prevalence studies to understand risks, and prepare resources– such as upscaled screening– to prevent increasing prevalence, clusters or outbreaks. Rapid molecular-based methods will be a crucial part of these considerations, as they can reduce unnecessary isolation and opportunity costs.

## Introduction

The rising prevalence of carbapenemase-producing Enterobacterales (CPE) poses a serious and severe threat to public health, leaving clinicians with fewer effective, low-toxicity, and affordable treatment options for infected patients. The incidence of CPE infection has increased across Europe and the UK over the last 20 years, causing significant concern [1, 2].

The purpose of this exercise was to bring together a panel of cross-specialty experts from England to discuss the barriers and challenges to implementing CPE guidance and to derive practical responses to questions frequently asked about screening for CPE. This is intended to provide support to clinicians and managers struggling to develop screening strategies, and attempt to ensure some consistency of approach while, at the same time, recognising the unique differences between institutions in terms of case-mix, demography, and geography.

## Methods

The experts were chosen from different specialities to reflect different relevant perspectives (clinical microbiology, infection prevention and control, laboratory microbiology, and hospital management) and different geographical areas within England. A review of the current global and national situation regarding CPE, as well as relevant local experience, was presented and then the panel sought to provide pragmatic responses to a set of six questions reflecting areas of uncertainty in implementing CPE screening. In view of the lack of strong scientific evidence for these responses, the panel members were asked to provide responses based on their experience and knowledge of the topics. Responses were collated and further discussed during three rounds of electronic review until a consensus was reached.

## Background

The ‘Big 5’ carbapenemase families (KPC, OXA-48-like, NDM, VIM and IMP) account for over 97% of CPE cases in England [2] and the most commonly identified host species are *K. pneumoniae*, *E. coli* and *Enterobacter* spp. England has more of a mixed carbapenemase epidemiological pattern across different geographic regions, compared to other European countries, and has also experienced more plasmid expansion due to horizontal transmission than clonal expansion [3]. Carbapenemases have been classified into classes A, B, and D, according to the Ambler classification [4, 5] and an understanding of this classification is needed to help clinicians decide the most appropriate therapeutic interventions [6], as phenotypic susceptibility can be predicted based on the presence or absence of a particular resistance mechanism. This is particularly true for β-lactam/β-lactamase inhibitor combinations, such as ceftazidime/avibactam [7], imipenem/relebactam and meropenem/vaborbactam, which are ineffective against metallo-β-lactamases, and for aztreonam, which is not active against serine enzymes.

## The burden of CPE

CPE colonisation and infection can lead to a number of adverse outcomes for operational flow and patient outcomes, with a high re-admission rates of up to 24% within 30 days of discharge [8] and a high 30-day mortality rate following infections, owing to limited therapeutic options [9]. A number of studies have demonstrated the relationship between prior carriage of CPE with subsequent infection, although the relative proportions of colonised to infected patients can vary [10, 11] Duration of CPE carriage varies significantly between patients but can be prolonged– from months to years [12]–which provides ample opportunity for both endogenous infection and transmission to others. This is especially true in healthcare settings, situations where antimicrobial selection pressure is a factor, or when high-risk invasive interventions are undertaken.

### Operational flow consequences

Effective prevention of CPE outbreaks has significant operational benefits, especially in the medium to long term, reducing the risk of incidents that disrupt patient flow due to bed or ward closure. This may require intensive screening and cohorting of contacts and instigating enhanced contact precautions. Failing to prevent outbreaks can cause logistical complications as isolation rooms or bays becoming limited, resources strained, and there may be significant additional burdens to healthcare staff. Previous studies have found that the length of stay of patients is greatly increased in patients with CPE compared to non-carriers [13]. Patient care is also often hindered by delayed or cancelled elective procedures, and the need for further therapeutic treatment.

### Financial consequences

As an antimicrobial resistant organism, the financial cost of managing CPE is also likely to be higher than other more susceptible infections, given the immediate need for isolation and the limited treatment options. These financial consequences are multifaceted with economic expenditure for longer hospital stays, enhanced infection control procedures, environmental interventions, opportunity costs and anti-infective treatments. One study estimated the median healthcare-related cost of treating a patient with a carbapenem resistant organism was more than double (£49,537 compared to £19,299) that of treating a patient with a sensitive organism [14]. The average daily cost of a stay in an isolation ward is £586 and the average intensive care unit (ICU) cost per day equals £1621.16 [15], so lengthened hospital stays can quickly increase unplanned economic expenditure. In addition, the limited antibiotic agents that are effective against CPE are typically more expensive, for example, cefiderocol, one of the only effective last-resort treatments for NDM, can cost up to £1,319 for 10 vials, which is considerably more than most first-line antibiotics [16]. Guidance from the National Institute of Care and Health Excellence (NICE) helped to bring this antibiotic to market as part of the NHS de-linkage scheme, a subscription-style payment model that aims to encourage the development of new antibiotics by paying manufacturers a fixed annual fee [17]. 

### Outbreaks costs

An outbreak of NDM-producing *K. pneumoniae* in 2017, which affected 40 patients across a group of five hospitals in West London, was estimated to cost €1.1 m over 10 months [13]. This was divided into €312,000 actual expenditure on resources for cleaning, enhanced CPE screening, isolation and monitoring of good hand and environmental hygiene practices, and €822,000 in lost “opportunity cost” based on staff time, bed closures and missed elective surgery reimbursements. In addition to the €1.1 million outbreak cost, €153,000 was also spent on estate expenditure prompted by the outbreak. Similarly, an outbreak of two strains of OXA-48 producing *K. pneumoniae* in the University Hospitals of Leicester NHS Trust was estimated to cost the organisation £350,000-400,000 for additional PPE, environmental decontamination, screening of contacts, treatment with ceftazidime-avibactam and staff costs [8]. The NHS is currently facing significant financial difficulty as well as lengthening waiting lists for treatment, and avoiding the cost of CPE outbreaks is important to help retain resources and avoid further ward closures.

## Methods to detect CPE

### Culture

Culture-based methods have been the cornerstone of microbiological investigation since the 19th century. However, application of these methods to CPE culture take on average 24–48 h to determine a positive result, or 48–72 h for the US Centers for Disease Control (CDC) method [18]. Preliminary positive results may then require further confirmation of carbapenemase production and identification of resistance genes. Changes to pathology services with the creation of Pathology Networks and adoption of an Essential Services Laboratory (hub-and-spoke) model have also led to some smaller hospitals losing the capability to perform culture-based methods. Delays in the transportation of samples between laboratories would be especially detrimental in the event of an outbreak or infection of high-risk patients. The sensitivity of culture-based methods can also vary between approaches and for each organism. For example, the reported sensitivity of MacConkey agar with carbapenem disks ranges from 75.8% to 96.9%, whereas the sensitivity of CDC broth incubation ranges from 65.6% to 98.8% [18]. 

### Molecular

Molecular methods, such as PCR, provide accurate, standardised, sensitive, and specific determination of the DNA and RNA of targeted microorganisms. These methods may offer fast turnaround times, which could ultimately save time and resources, as well as preventing clinical infections, by implementing infection control interventions earlier [19]. However it should be noted that molecular methods can miss low-prevalence carbapenemases so where appropriate should be used in conjunction with culture-based techniques, especially for diagnostic testing. The return on investment from faster turnaround times and reduced length of hospital stays has the potential to outweigh the unit cost of molecular testing, with one study reporting a £462 per patient cost saving for a five-day hospital stay after replacing culture-based screening with PCR testing for CPE [20]. An approach based on molecular testing using Cepheid’s Xpert® Carba-R test was also shown to reduce the mean number of bed-days lost per month from 22.8 to 9 days compared to culture-based screening [19]. 

## Current UK guidelines for managing CPE

In 2020, Public Health England (PHE)– now the United Kingdom Health Security Agency (UKHSA)– published a framework of actions to contain CPE, which was later updated in September 2022 [21]. This guidance emphasised the importance of each healthcare provider establishing its own active admission screening based on regional prevalence, patient mix and links to other healthcare providers, and recommended all Trusts implement molecular or immunochromatographic assays for the detection of KPC, OXA-48-like, NDM and VIM carbapenemases. This was accompanied by an update to the Health Protection Regulations in 2020, making it a requirement to report CPE cases to PHE notifications of infectious diseases (NOID) [22], to enable the publication of detailed surveillance reports on the rate of infections in the UK [23]. 

While this guidance is extremely helpful, there are still many constraints, financial barriers and practical issues to address when designing a local active surveillance and infection prevention programme. It is prudent to address these at an early stage as CPE rates continue to rise, and experience from the COVID-19 pandemic regarding screening, isolation and establishing cohorts could potentially be translated. Healthcare providers are also grappling with the need to implement cost improvement plans as NHS funding is under pressure. This puts proactive actions that could save money ahead of reactive actions, and screening is the only way to truly understand the extent of prevalence, prevent or manage outbreaks, monitor local epidemiology and understand the risk factors [24]. 

Managing patients with CPE in a hospital setting requires professionals in a range of specialties, including infection prevention and control nurses, critical care specialists, pharmacists, laboratory specialists– such as microbiologists, clinical scientists, and biomedical scientists– and members of the hospital boards who ultimately need to make investment choices based on priorities. In addition, the role of all involved healthcare staff, especially nursing and cleaning staff on wards, is essential to successful rollout of a screening programme.

## What are the primary strategic and operational challenges for implementing the framework?

Recently established integrated care systems in the UK (ICSs) are currently grappling with a number of competing priorities partially induced by the COVID-19 pandemic, including returning to and increasing the delivery of pre-pandemic elective care activity [25, 26], funding pressures [27], workforce shortages across different departments [28] and avoiding prolonged waits for unplanned admissions [29]. These come on top of existing NHS hospital estate infection prevention challenges including the shortage of ensuite single occupancy patient rooms that are essential for effective source isolation [30]. Healthcare-associated CPE infections will only exacerbate delays, ward closures and cancelled operations. However, dealing with CPE is often neglected in favour of more immediate priorities and the long-term consequences are not being fully considered. Better understanding of local epidemiology and the impact of a potential outbreak will help to demonstrate the critical need for a framework for CPE testing, which is vital for reducing risk to lives and more efficient use of resources.

Organisations that do not already have clear protocols for screening for CPE should assess the resource and cost implications of sampling, laboratory testing, pre-emptive and focussed isolation or cohorting, data collection, education and training. These settings also need to understand when it is appropriate to start screening based on risk and the potential consequences of inaction. Developing these protocols should include co-operation from many different departments across an organisation, including likely approval by healthcare provider management teams and emerging ICS structures. The protocol that is established must reflect the UKHSA framework, which itself must be kept current, as well as balance the needs, demands and agendas of each department and their competing priorities, and still be adaptable to change. However, it is only possible to do this effectively once the need is communicated through education and awareness. The logistics of how a protocol may be implemented must also be considered; introducing standardised data collection and analysis is key to identifying high-risk patients, as well as ensuring data is easily accessible, reducing the burden on frontline staff, and creating robust and deliverable information that is required for surveillance.

## What learning can be leveraged from the approaches used during the COVID-19 pandemic?

The COVID-19 pandemic put institutions under unprecedented pressure to quickly establish new protocols. The upside of this is that clinical staff, at all levels, will understand the reason for the delivery of implementing screening and isolation for vast numbers of patients; everyone now knows how quickly infections can spread and the importance of early control. Language such as PCR, R-rates and transmission are no longer alien to non-medical individuals.

COVID-19 highlighted how effective infection control protocols are imperative to maintaining clinical flow and operational capacity. This required improvements in logistics, reporting and communication within and between departments, as well as the breaking down of traditional boundaries between different staff groups. The pandemic also accelerated the digital journey with innovations such as track and trace and reporting vaccinations on the NHS app. These lessons ultimately stemmed from a nationwide command and control approach; providing policies and resources as well as clear expectations from the top down helped to galvanise action to be taken at a local level.

The need to optimise the use of resources also transformed the understanding of outbreaks from being purely about health, to including economic consequences and the indirect impact on patient care. Fiscal pressures became a big driver for the pandemic response, as departments were forced to close, elective procedures were postponed, and staff resources were limited. Cost-improvement strategies are now high on the agenda for healthcare provider boards as they seek to recover from the impacts of the pandemic.

Throughout the COVID-19 pandemic, PCR testing emerged as the superior assay format in terms of accuracy and sensitivity, forming the backbone of nationwide screening regimens [31]. The majority of NHS laboratories now have the capability, awareness and potentially even the infrastructure to carry out molecular testing, so that shifting from culture- to molecular-based workflows to accelerate turnaround times is not the jump it might have been before.

Some institutions have already reviewed lessons learnt from the pandemic [32], providing valuable insight into future challenges and guiding them as they plan new programmes. However, there is no ‘one size fits all’ approach to preventing the transmission of an infectious disease. The diversity of CPE transmission routes (patient-to-patient, healthcare workers, healthcare equipment and water environment) and mobility of resistance genes pose different challenges to those encountered with COVID-19, and CPE outbreaks are likely to span a longer time course, creating the risk that further spread of resistance will render certain antibiotics obsolete.

## Who are the most important high-risk patients or settings for priority screening?

Each healthcare provider should conduct its own risk assessment based on regional prevalence, local understanding of risk factors, patient mix and links with other care providers. The following groups may be considered for prioritisation for screening (Table 1):

**Table 1:** Schematic representation of a suggested screening approach Recommendations for the actions to be taken, and the role of molecular screening, for four high-risk groups that should be prioritised for CPE screening in healthcare settings.

Type of screening	Action to be taken	Role for rapid molecular testing
Active surveillance of high-risk unplanned admissions	Risk assess patients based on epidemiological history Pre-emptively isolate highest-risk patients If screening results are **positive**: • Use molecular or immunochromatographic testing to identify resistance mechanisms where necessary • Negative results may be used to de-escalate isolation	To rapidly determine carriage status and requirements for isolation: For CPE **negative** cases: • Improve patient flow • Retain bed capacity • Prevent unnecessary isolation protocols or empirical treatment For CPE **positive** cases: • Instigate infection control procedures • Prevent transmission and outbreaks • Consider clinical impact if infection present e.g., alteration to empiric therapy pending further results
Pre-admission screening for high-risk elective patients	Risk assess patients based on epidemiological history Determine whether there is a need to isolate patients or to establish cohorts If results are **positive**: • Further risk assessment for the procedure required based on probability of post operative infection • Risk assess whether targeted prophylactic and/or empiric antibiotic therapy indicated	Consider, if: • The procedure is imminent, and results are needed urgently; • There is a lack of laboratory resources or staffing for culture-based methods.
Regular screening of patients on high-risk units	Use as a sentinel surveillance method in addition to other screening strategies, and protect highest risk settings. If results are **positive**: • Isolate positive high-risk patients to avoid transmission of CPE	Consider, if: • There is a lack of laboratory resources or staffing for culture-based methods.
Screening of contacts of known cases	If results are **positive**: • Urgent identification and isolation of known contacts • Instigate infection control cleaning protocols	Yes– the faster turnaround time is important for preventing further transmission, and retaining resources and beds in large outbreaks.

1. High-risk unplanned admissions.
2. High-risk elective admissions.
3. Patients in high-risk units, such as augmented care (e.g., critical care, dialysis or transplant units).
4. Contacts of known cases prior to appropriate isolation or in the case of clusters or outbreaks.

Local arrangements should consider ease of implementation of selection of patient screening protocols. Criteria for screening that use a limited number of readily answered questions may be preferred to more accurate but less accessible variables.

High-risk patients should ideally be presumptively isolated while they wait for CPE screening results.

However, this is not feasible when there are many cases or limited single rooms and resources, so clinicians need to make informed decisions and prioritise. Rapid molecular testing can help rule out colonisation in patients testing negative and free up limited isolation bed capacity. Screening of these patients is also important for ongoing epidemiological surveillance and results should be regularly reported to the UKHSA and relevant institutional leads for further action where necessary, creating a feedback loop to develop and modify screening protocols.

## Who are the most important high-risk patients for priority pre-emptive isolation?

It is not always realistic to pre-emptively isolate all patients pending results of CPE screening, so it is important to identify those most at risk. The group agreed that the criteria for these patients differ between hospitals; there are no set rules, but several options were discussed:

- Those who have travelled from overseas or are transferred from an institution with a known high prevalence of CPE in the preceding year or are known previous CPE carriers are recommended for screening,
- Admissions to high-risk units, such as augmented care (e.g., critical care, dialysis or transplant units).
- Vulnerable or immunocompromised patients who frequently visit either the same hospital or multiple centres, such as transplant or renal dialysis patients.
- Gastrointestinal/hepatobiliary surgery patients at high risk of intra-abdominal infections.

Once admitted, patients may be screened either weekly or monthly to determine point prevalence and to better define clinical areas linked to higher rates of CPE transmission.

## What is the role of rapid molecular PCR screening for CPE, and for which high-risk patients/settings?

Rapid molecular PCR testing becomes of enhanced value when urgent results are needed for decision making with regards to isolation, cohorting, individual patient management– such as preventing further delays to or risk from invasive procedures– and closing beds/wards to new admissions. Easy-to-use, rapid molecular tests prove the best approach for streamlining high-volume testing workflows in the laboratory and returning results quickly, compared to manual culture or immunochromatographic methods. This can help to reduce the burden of unnecessary isolation and provides earlier clarity than culture for infection control [19]. PCR screening can run several investigations at once, which is particularly beneficial where microbiology staff and resourcing are limited. These methods do not rely on a traditional laboratory set-up so can be implemented in decentralised settings, which would improve turnaround times further by reducing transportation delays.

Screening for the resistance gene can also help Trusts to identify the carbapenemase responsible for an outbreak and understand the mode of resistance. This is not only beneficial when reporting a positive case to the UKHSA, but also for directing treatment and quickly ruling out potentially inappropriate antimicrobials. The class of carbapenemase can provide insight into the susceptibility to some β-lactam inhibitor combinations, although phenotypic susceptibility testing is also essential. However, it should be borne in mind that a limitation of all target-specific PCR tests is that non-target gene sequences will not be detected and so carbapenemase genes not included in the test repertoire will be missed. While this appears to be a minor issue in the UK currently, it is likely that carbapenemase genes more usually seen in non-Enterobacterales, e.g. non-OXA-48 oxacillinases, will be missed and this issue should be kept under review.

## What are the evidence requirements at a healthcare provider and/or pathology network level needed to support new and sustained laboratory investment that will enable new screening recommendations?

There is already a wealth of evidence demonstrating increasing prevalence of CPE worldwide and in the UK, the role of hospitals as key locations where transmission is amplified, and the value of active surveillance for both patient throughput and minimising transmission and outbreaks. However, the new UKHSA framework is heavily reliant on each healthcare provider implementing risk assessments based on essential surveillance, intelligence and regional prevalence. Therefore, it is imperative that up-to-date and on-going local prevalence surveys with data on risk factors and likely acquisition pathways are conducted across the UK to fully understand the risk of colonisation or infection and to help decision makers agree on a screening strategy and know whether to scale up or down operations.

Understanding more about the transmission of CPE will also educate decision makers on which patient cohorts are most at risk and on the best practice for screening. Currently, there is limited good quality evidence to identify best practice approaches to preventing CPE transmission. Prevention of outbreaks by pre-emptive isolation while waiting for screening results was supported by a prospective non-randomised study [33], but more robust experimental designs, especially cluster randomised trials, are needed to account for the impact of interventions on same-institution patients [34]. Arming healthcare providers with information on transmission rates, infectious periods, colonisation-to-infection conversion rates, and consequences of colonisation and infection can help to determine urgency and improve the efficacy of interventions.

Further information regarding the impact of CPE positive cases on the operational flow of a hospital– including cancellation of procedures, closing beds or wards, staffing levels and resources– with estimations of the financial consequences would be beneficial to fully conceptualise the cost implications of outbreaks as well as endemic infection. This is an area that requires much more work. The impact of disrupted services on the quality of patient care also needs to be assessed to understand the severity of outbreaks and cases beyond initial infection, morbidity and mortality rates. This can be determined on both a Trust-wide scale and an individual patient basis, shining light on the impact to patient management and outcomes. An example of this was a study conducted at the University Hospital of Leicester NHS Trust, which recorded both the financial cost and the detrimental impact of a CPE outbreak on the quality of patient care, including mortality rate [8]. However, the financial implications of managing CPE patients can be significant outside of an outbreak setting, totalling more than double the costs of managing non-colonised/infected patients [14]. 

The role that molecular testing plays in helping to limit the financial burden of CPE, maintain patient flow and improve treatment outcomes by reducing the turnaround times for screening results could also be further highlighted in case studies. For example, Corless et al. expressed the tangible benefit of molecular testing in reducing the number of bed-loss days, which translated into improved patient flow and more efficient use of resources [19]. 

## Conclusions

The alarming escalation of CPE rates across the world, and the heterogeneity of organisms and resistance genes in the UK is a dire concern that cannot be ignored. This position paper brought together experts in the field to consider six of the most pressing questions regarding the implementation of CPE recommendations to support healthcare providers and ICSs as they make informed decisions about screening in line with the UKHSA framework. Screening for CPEs aims to identify carriers with the intention of enabling targeted interventions to prevent infection in carriers and transmission to others. The value of screening is determined by the effectiveness of the interventions as well as by the availability of other actions that can mitigate the harm accompanying colonisation or infection. If interventions are of little use, or if effective treatment of infections caused by CPEs is readily and cheaply available, then screening may serve minimal purpose. However, there is evidence of the effectiveness of isolation of CPE carriers as a transmission prevention intervention (e.g. [33]), and treatment of CPE infections with ceftazidime-avibactam and cefiderocol is expensive and already compromised by reports of resistance, especially amongst NDM-producing CPE isolates [35]. Consequently, there is a *prima facie* role for screening in tackling CPEs.

Expert opinion across the board was that decisive and instructive information is needed to determine how to establish CPE screening protocols, and molecular-based methods have a significant role to play. Molecular methods prove crucial in any instance where time is of the essence, especially when there is a high number of cases, limited staffing or laboratory capacity, disruption to patient flow, outbreak risk and immediate concern for patient health. There are substantial differences between the capabilities of different healthcare providers and the risk of an outbreak, so no ‘one size fits all’ blanket approach would work for establishing screening protocols. Further research into local epidemiology and the consequential counterfactuals of not instigating a screening protocol will help to highlight the need for robust policies at each healthcare provider.

## Data Availability

No datasets were generated or analysed during the current study.

## Abbreviations

CPE: Carbapenemase-producing Enterobacterales
UK: United Kingdom
NHS: National Health Service
UKHSA: UK Health Security Agency
ICS: Integrated Care System
KPC: Klebsiella pneumoniae carbapenemase
OXA: Oxacillinase
NDM: New Delhi metallo-β-lactamase
VIM: Verona integrated-encoded metallo-β-lactamase
IMP: Imipenemase
spp: Species
ICU: Intensive care unit
NICE: National Institute of Care and Excellence
CDC: Centers for Disease Control
PCR: Polymerase chain reaction
DNA: Deoxyribonucleic acid
RNA: Ribonucleic acid
PHE: Public Health England
NOID: Notifications of infectious diseases
R-rate: Reproduction rate
